# Advantages and limitations of commercially available electrocuting grids for studying mosquito behaviour

**DOI:** 10.1186/1756-3305-6-53

**Published:** 2013-03-07

**Authors:** Silas Majambere, Dennis J Massue, Yeromin Mlacha, Nicodem J Govella, Steven M Magesa, Gerry F Killeen

**Affiliations:** 1 Ifakara Health Institute, Kiko Avenue, Mikocheni, P. O. Box 78373, Dar es Salaam, Tanzania; 2 National Institute for Medical Research, Amani Medical Research Centre, P. O. Box 81, Muheza, Tanzania; 3 Liverpool School of Tropical Medicine, Pembroke Place, Liverpool, L3 5QA, UK; 4 RTI International, Centre for Strategic Malaria Solutions, Global Health Group, P. O. Box 1181–00621, Nairobi, Kenya

**Keywords:** Electrocuting grids, Human landing collections, Mosquito, Malaria, Behaviour

## Abstract

**Background:**

Mosquito feeding behaviour plays a major role in determining malaria transmission intensity and the impact of specific prevention measures. Human Landing Catch (HLC) is currently the only method that can directly and consistently measure the biting rates of anthropophagic mosquitoes, both indoors and outdoors. However, this method exposes the participant to mosquito-borne pathogens, therefore new exposure-free methods are needed to replace it.

**Methods:**

Commercially available electrocuting grids (EGs) were evaluated as an alternative to HLC using a Latin Square experimental design in Dar es Salaam, Tanzania. Both HLC and EGs were used to estimate the proportion of human exposure to mosquitoes occurring indoors (π_i_), as well as its two underlying parameters: the proportion of mosquitoes caught indoors (P_i_) and the proportion of mosquitoes caught between the first and last hour when most people are indoors (P_fl_).

**Results:**

HLC and EGs methods accounted for 69% and 31% of the total number of female mosquitoes caught respectively and both methods caught more mosquitoes outdoors than indoors. Results from the gold standard HLC suggest that *An. gambiae s.s.* in Dar es Salaam is neither exophagic nor endophagic (P_i_ ≈ 0.5), whereas *An. arabiensis* is exophagic (P_i_ < < 0.5). Both species prefer to feed after 10pm when most people are indoors (P_fl_ > > 0.5). EGs yielded estimates of P_i_ for *An. gambiae s.s., An. arabiensis* and *An. coustani*, that were approximately equivalent to those with HLC but significantly underestimated P_fl_ for *An. gambiae s.s.* and *An. coustani.* The relative sampling sensitivity of EGs declined over the course of the night (p ≤ 0.001) for all mosquito taxa except *An. arabiensis.*

**Conclusions:**

Commercial EGs sample human-seeking mosquitoes with high sensitivity both indoors and outdoors and accurately measure the propensity of Anopheles malaria vectors to bite indoors rather than outdoors. However, further modifications are needed to stabilize sampling sensitivity over a full nocturnal cycle so that they can be used to survey patterns of human exposure to mosquitoes.

## Background

To design effective malaria vector control interventions, it is imperative to understand the behavioural ecology of mosquitoes [1-3]. Although mosquitoes have been known to be the vectors of malaria for centuries there are still major gaps in the knowledge base regarding mosquito behaviour as they seek for blood and sugar meals, mating opportunities and breeding sites. To assess blood feeding behaviour of human-seeking female mosquitoes, the only method that has been widely accepted as gold standard for measuring human exposure rates to bites is the human landing collection (HLC) [4]. This technique can be used both indoors and outdoors to assess where and when people are exposed to mosquito bites [5]. However, this method poses serious ethical concerns [6] as it exposes human participants to mosquito bites that are potentially infected with malaria and a diversity of other vector-borne pathogens. Moreover, it is difficult to supervise, labour intensive and requires considerable and consistent skill in catching mosquitoes over long periods of the night [4,7].

Many alternative methods have been developed and evaluated as potential replacements for the HLC. The Centre for Disease Control (CDC) miniature light traps (LT) [8-10], are widely used but are far more efficient indoors than outdoors. Pyrethrum spray catch (PSC) is also widely used but this method only samples indoor-resting mosquitoes [11] and underestimates mosquito biting densities in houses where use of irritant or repellent insecticides deter resting indoors after feeding [12] and also in places where mosquitoes are endophagic but exophilic [2]. The Mbita bednet trap seemed to exhibit high sensitivity for catching human-seeking mosquitoes indoors in rural Kenya [13-15] but this could not be replicated in the highlands of Madagascar, with a highly zoophilic mosquito population [16], and in Northern and South Eastern Tanzania [17,18]. Resting boxes [19,20] or claypots [21] are sometimes used outdoors to collect outdoor resting mosquitoes. The Furvela tent traps and Ifakara tent traps [5,7,19] also collect human-seeking malaria vector mosquitoes but it is unclear whether mosquito samples obtained with these devices represent endophagic or exophagic mosquito populations or a mixture of the two [22]. Moreover, tent traps and light traps are inconsistent in terms of their relative trapping efficiency across seasons and locations [7,23].

All these trapping techniques might be useful in isolated contexts but are not reliable and consistent enough to conduct quantitative surveys of where and when mosquito-human interactions occur through blood feeding. A particularly useful indicator of mosquito-human behavioural interactions, that can be used to predict the impact of domestic vector control measures such as long lasting insecticidal nets (LLINs) or indoor residual spraying (IRS) [24-26], is the proportion of human exposure to mosquito bites which occurs indoors (π_i_) [5,27]. A particular requirement of a mosquito sampling method to be used for estimating this quantity is that it must have the same sensitivity, relative to HLC, indoors and outdoors and across all periods of the night. As of yet, we are not aware of any evaluation of any mosquito trap that satisfies this requirement and can therefore replace HLC.

Electrocuting grids (EGs) have long been used for sampling tsetse flies (Diptera: Glossinidae) [28] and mosquitoes. They have been used to study tsetse fly behaviour, such as their response to odour [29], and flight behaviour [30]. Electric nets have also been used in studying the feeding preference of flies [31]. In addition, the method has been modified for trapping mosquitoes and studying their flight behaviour as they approach an attractive host [32,33] as well as their host preferences [33-35]. In fact, EGs were deemed more effective at trapping mosquitoes close to odour sources than CDC light traps or entry traps [33].

Outside these research areas, this principle has been widely used in trapping a variety of flying insects that are attracted to light [36]. In many food handling and recreational centres, commercially available devices are commonly used that use strong fluorescent light sources to attract mosquitoes and other flying insects to EGs to reduce pest nuisance to humans and domestic animals [37].

Unlike the custom-made devices used strictly for research applications as described above, the commercially manufactured and widely available EGs have not been previously evaluated as tools with which to study mosquito host-seeking behaviour. Torr and colleagues concluded their paper with this remark: “…electrocuting technology, largely in its present form, opens important routes to an essential topic: the fuller understanding of mosquito behaviour” [33]. Therefore, this study aims to test the potential for commercially manufactured EGs to monitor mosquito biting behaviour both indoors and outdoors to determine whether it could reliably replace the gold standard HLC method as a means to conduct quantitative surveys of mosquito biting activity and human exposure to it.

## Methods

### Study area

The study was performed in the Jangwani ward along the Msimbazi River valley in Dar es Salaam, the largest city in the United Republic of Tanzania. The city is situated on the shores of the Indian Ocean with a hot and humid climate, which is ideal for mosquito proliferation and malaria transmission. There are typically two rainy seasons: a main rainy season from March to June and a more erratic, less intense rainy season from October to December. The study was carried out from May to June 2010.

### Experimental design

**Figure 1:**
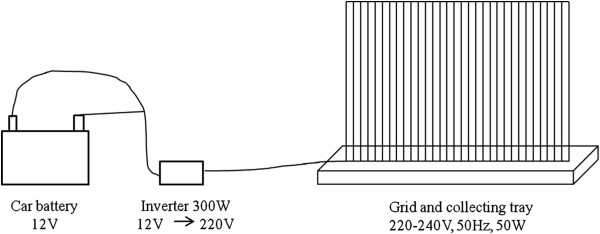
**Electric grid set up.** The figure is a schematic representation of the electrocuting grid, its tray for insect collection, powered by an inverter connected to a car battery.

**Figure 2:**
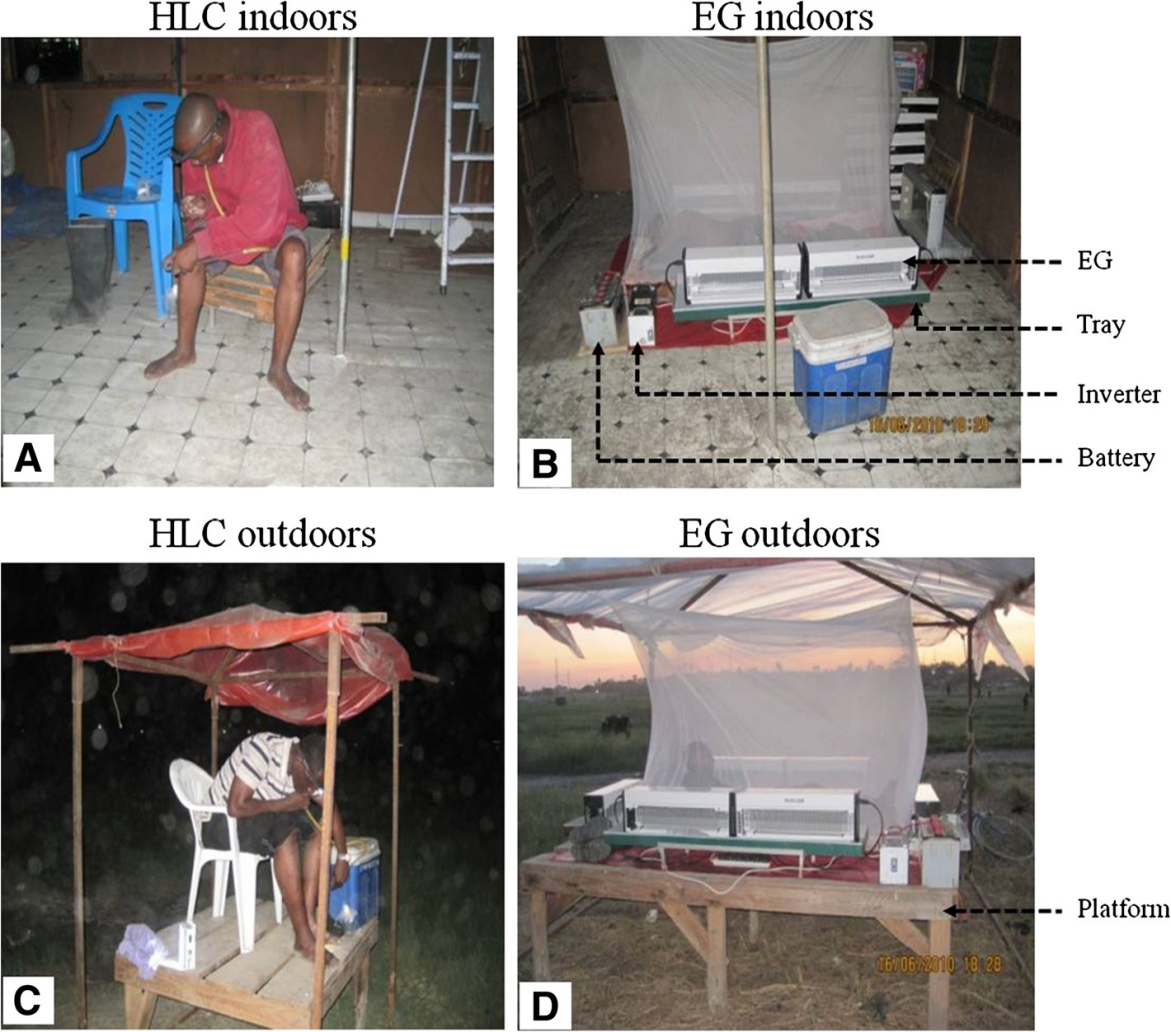
**Experimental set up.** The figure shows a volunteer performing human landing collection indoors (**A**); a volunteer sleeping under a bednet, surrounded by electrocuting grids indoors (**B**); a volunteer performing HLC outdoors on a raised and sheltered platform (**C**); a volunteer sleeping under a bednet outdoors on a raised and sheltered platform (**D**).

Four Ifakara design experimental huts [38] were built in an open field in the river flood plain, 50m away from human settlements and the huts were placed at 50m intervals to minimize interference between these experimental huts and existing human dwellings. Although these huts are based on representative housing dimensions in the Kilombero Valley, they are also similar to common housing structures found in Dar es Salaam. The EGs used were of the PlusZap™ model ZE107 PZ40W (http://insect-o-cutor.co.uk/telerikfiles/Insect-O-Cutor%20Catalogue%20300112%20-%20PlusZap.pdf, P + L Systems Ltd, Knaresborough, UK) and were 0.68 × 0.15 × 0.24 m in size. Mosquitoes could be caught flying through the grids from either side. Before use, the fluorescent light tubes were removed to prevent interference with the natural stimulus of the human volunteer used as bait. For the EGs experiment, each human bait slept on a mattress, was protected by an untreated polyester bed net and surrounded by six grids connected to a fully charged 12V car battery via an inverter which converts this direct current to 220V 50Hz alternating current (Figure 1). Indoor experiments were performed inside the experimental huts while the outdoor experiments were performed on a wooden platform 20m away from the experimental huts in the opposite direction to human dwellings. The platforms and huts were raised 50cm off the ground to avoid flooding in the rainy season (Figure 2).

The platforms were roofed with nylon sheeting to protect the participant and equipment from rain. Mosquitoes that were killed or stunned after colliding with the grids, fell into 1.4 × 0.35 × 0.06 m collection trays in which the grids were placed and were emptied every hour. While the participants in EGs experiments slept safely under intact untreated bed nets, those who performed HLC worked for 45 minutes of each hour, with the remaining 15 minutes used to rest and also collect mosquitoes from the collection trays.

There were 4 pairs (8 in total) of experimental units, each consisting of one pair of volunteers sleeping surrounded by EGs (one volunteer indoors and the other outdoors at a single experimental hut) and a corresponding pair of volunteers conducting HLC (one indoors and one outdoors at the other experimental hut). These 2 × 4 sets of EGs and HLC catches were assigned alternatively to the eight individually numbered, experimental units. Each night, the alternating methods were moved consistently from one experimental unit to the next experimental unit so that all 4 sets of grids were rotated through the entire set of experimental units every 8 nights. Two catchers were allocated to and remained associated with a particular experimental unit but rotated between the indoor and outdoor stations every two nights to avoid covariance of the rotation of their arrangement with the rotation of the two methods every night. Experiments were performed between 6pm and 6am in all experimental units.

All mosquitoes collected were transported to the laboratory every morning, counted and identified morphologically to genus (*Culex*, *Aedes*, *Mansonia* and *Coquillettidia spp*.), species group (*An. funestus sensu lato*) or species complex (*An. gambiae s.l.* and *An. coustani s.l.*) [39,40]. *An. gambiae s.l.* species were stored individually in eppendorf tubes containing silica gel and sealed properly to keep the samples dry. These samples were sent to the Ifakara molecular laboratory for species identification using polymerase chain reaction (PCR) [41].

### Data analysis

The cut off point at which most people in Dar es Salaam have moved indoors was estimated to be 10pm by questionnaire surveys [42]. The same surveys revealed that most people remained in their houses until after 6am when HLC surveys had terminated. Therefore, the night was broken into two intervals, before and after 10pm, with the assumption that humans are exposed outdoors before 10pm and indoors after 10pm. Three outcomes were estimated by these two methods to compare their ability to quantify mosquito feeding behaviours and the interactions between mosquitoes and humans that influence disease transmission [27]: (1) the proportion of all mosquitoes caught that were captured indoors (*P*_*i*_) was obtained by dividing the number of mosquitoes caught indoors by the same number plus the number of mosquitoes caught outdoors. (2) The proportion of all mosquitoes caught that were captured when most people are indoors (*P*_*fl*_) was obtained by dividing the number of mosquitoes caught when most people are indoors divided by the same number plus the number of mosquitoes caught before most people retire indoors (3). The proportion of human exposure that occurs indoors (π_i_) was calculated by dividing the number of mosquitoes caught indoors during the period that most people are indoors by itself plus the number of mosquitoes caught outdoors outside of that period. *P*_*i*_ and *P*_*fl*_ are considered underlying determinants of π_i_.

These crude binary estimates of *P*_*i*,_*P*_*fl*_ and π_i_ allowed statistical comparisons by logistic regression using generalized linear models (GLM) with a binomial distribution and a logit link function [27,43]. Every sampling night started at 6pm so that the first sampling hour was completed at 7pm, and was terminated at 6am, corresponding with the 12^th^ sampling hour. To test whether the sensitivity of EGs in estimating different outcomes relative to HLC declined with time, logistic GLMs were fitted with time (in intervals of one hour from the start of collections) as a continuous independent covariate and the proportion of captured mosquitoes which were caught by the grids (EG/(EG + HLC)) as the dependent variable so that an outcome of 0.5 represents equivalence of the two methods. All data were analysed using PASW (formerly SPSS) version18.0.

### Ethical consideration

Permission to carry out this study was obtained from the Institutional Review Board of the Ifakara Health Institute (IHI), the Medical Research Coordination Committee (MRCC) of the National Institute for Medical Research (NIMR, Reference number: NIMR/HQ/R.8a/Vol. IX/279) and the Ethics Review Panel of the Liverpool School of Tropical Medicine (LSTM). Informed consent was provided by all participants involved in the study.

Participants were provided with the antimalarial prophylaxis Malarone® (atovaquone proguanil) and were screened weekly by a specially trained laboratory assistant for malaria parasites with both rapid diagnostic tests and microscopy. None was diagnosed as positive for a blood-stage malaria infection, but if they had, these participants would have been provided free of charge the standard front-line malaria treatment in Tanzania, namely Co-Artem® (Artemether-Lumefantrin). In the case of withdrawal of any participant prior to completion of the study, replacement was made as soon as possible with a new recruit continuing with the field experiment as described above.

Permission to publish these findings was obtained from the National Institute for Medical Research (NIMR, Reference number: NIMR/HQ/P.12 VOL.XIII/10).

## Results

A total of 168,526 female mosquitoes were collected, of which 97% (164,145) were *Culex* species. Of the 2,728 Anopheles mosquitoes caught, 80% (2,214) were of the *An. coustani* complex and 20% (568) of the *An. gambiae* complex. These three taxa were included in the subsequent analyses. Other species caught in very low numbers included *An. funestus*, Aedes, Mansonia and Coquillettidia species altogether comprising only 0.6% of the total catch. The two methods caught overwhelmingly more female mosquitoes than males, which accounted for only 2% of the total and were not included in subsequent analyses. The HLC and EG methods accounted for 69% and 31% of the total number of mosquitoes caught, respectively. All 568 mosquitoes morphologically identified as *An. gambiae s.l.* were subjected to PCR for species identification and 80% of these successfully amplified and were identified to species level: 315 were *An. gambiae s.s.* and 139 *An. arabiensis*.

The results presented in Table 1, suggest that *An. gambiae s.s.* in Dar es Salaam is neither exophagic nor endophagic (*P*_*i*_ ≈ 0.5) but shows a clear preference to feed after 10pm when most people are indoors (*P*_*fl*_ > > 0.5) and consequently the proportion of human exposure occurring indoors is high for this species (π_i_ > > 0.5). *An. arabiensis* in this area shows an exophagic behaviour (*P*_*i*_ < < 0.5) but prefers to feed at times when most people are indoors (*P*_*fl*_ > > 0.5) and in this case human exposure indoors is neither low nor high (π_i_ ≈ 0.5).

**Table 1:** Proportion of mosquitoes caught indoors, during sleeping hours and human exposure estimates

**Taxon**	**Method (N)**	**Proportion caught indoors (P**_**i**_**)**		**Proportion caught when most humans are indoors (P**_**fl**_**)**		**Proportion of human exposure occurring indoors (π**_**i**_**)**	
		^**1**^**Estimate [95% CI]**	**p**	**Estimate [95% CI]**	**p**	**Estimate [95% CI]**	**p**
*An. gambiae*	EG (122)	0.459 [0.373-0.548]	0.366	0.631 [0.542-0.712]	0.004	0.612 [0.471-0.737]	0.119
	HLC (193)	0.518 [0.448-0.588]	0.614	0.855 [0.798-0.898]	<0.001	0.853 [0.770-0.909]	<0.001
*An. arabiensis*	EG (36)	0.306 [0.178-0.472]	0.023	0.722 [0.556-0.844]	0.010	0.529 [0.303-0.745]	0.808
	HLC (103)	0.311 [0.229-0.406]	<0.001	0.748 [0.655-0.822]	<0.001	0.565 [0.421-0.700]	0.378
*An. coustani*	EG (295)	0.268 [0.221-0.321]	<0.001	0.685 [0.629-0.735]	<0.001	0.435 [0.345-0.530]	0.179
	HLC (1919)	0.222 [0.204-0.241]	<0.001	0.773 [0.754-0.791]	<0.001	0.492 [0.452-0.531]	0.686
*Culex spp*	EG (51995)	0.321 [0.317-0.326]	<0.001	0.629 [0.625-0.633]	<0.001	0.446 [0.440-0.452]	<0.001
	HLC (112150)	0.375 [0.372-0.378]	<0.001	0.694 [0.692-0.697]	<0.001	0.572 [0.568-0.576]	<0.001

EG: electrocuting grids; HLC: human landing catch; CI: confidence intervals.

Both *An. coustani* and *Culex spp* were exophagic and preferred to feed at times when most people are indoors. Although there seems to be no high or low indoor human exposure to *An. coustani*, it is significantly high for *Culex spp*.

There was no statistically significant difference between EGs and HLC in estimating *P*_*i*_ (p = 0.307), however, EGs significantly underestimated *P*_*fl*_ (p < 0.001) and π_i_ (p = 0.001) compared to HLC. For *An. arabiensis*, there was no significant difference between EGs and HLC in estimating all the three outcomes. For *An. coustani*, there was no significant difference between EGs and HLC in estimating *P*_*i*_ and the overall π_i_, but EGs significantly underestimated *P*_*fl*_ (p = 0.001). As for *Culex spp*, EGs significantly underestimated all the three outcomes compared to HLC (Table 2).

**Table 2:** Potential of electrocuting grids to predict different outcomes compared to the HLC gold standard

**Taxon**	**Method (N)**	**Proportion caught indoors (P**_**i**_**)**		**Proportion caught when most humans are indoors (P**_**fl**_**)**		**Proportion of human exposure occurring indoors (π**_**i**_**)**	
		^**2**^**OR [95% CI]**	**p**	**OR [95% CI]**	**p**	**OR [95% CI]**	**p**
*An. gambiae*	EG (122)	0.789 [0.501-1.243]	0.307	0.290 [0.169-0.500]	<0.001	0.272 [0.123-0.602]	0.001
	HLC (193)	1		1		1	
*An. arabiensis*	EG (36)	0.976 [0.429-2.223]	0.954	0.878 [0.374-2.036]	0.765	0.865 [0.283-2.643]	0.800
	HLC (103)	1		1		1	
*An. coustani*	EG (295)	1.282 [0.969-1.695]	0.081	0.639 [0.489-0.835]	0.001	0.796 [0.527-1.202]	0.278
	HLC (1919)	1		1		1	
*Culex spp*	EG (51995)	0.789 [0.772-0.807]	<0.001	0.747 [0.731-0.764]	<0.001	0.602 [0.583-0.620]	<0.001
	HLC (112150)	1		1		1	

^2^ OR: odds ratio.

Furthermore, we tested whether the sensitivity of EGs relative to HLC declined with time through the night. Results show that for *An. gambiae s.s.*, EGs caught more mosquitoes during the early hours of the night but their sensitivity dropped continuously through time until midnight when it reached a third of that of HLC and remained around that value until morning (Figure 2A). For *An. arabiensis*, EGs had approximately one quarter of the sensitivity of HLC but there was no clear pattern of increase or decrease through the night, although there was a poor fit for the model (Figure 2B), perhaps due to the very low numbers of samples caught for this species. For *An. coustani* EGs had approximately two thirds the sensitivity of HLC at the start of the night and then exhibited a slight but steady decline as the night progressed (Figure 2C). In the case of *Culex spp* EGs estimates were consistently lower than HLC and this relative sensitivity differed between indoor and outdoor stations (Figure 2D). Although EGs sensitivity relative to HLC seemed to drop overnight indoors, it remained constant through the night while sampling outdoors (Figure 2D).

## Discussion

The findings reported here show that commercially manufactured electrocuting grids can be used for monitoring mosquito behaviour and their interactions with human hosts. It can be used to sample mosquitoes indoors and outdoors efficiently, something most traps cannot do. The most encouraging finding of this study is that EGs appear to approximate HLC estimates in terms of the propensity of *Anopheles* taxa to feed indoors rather than outdoors (P_i_). This study was performed within a framework of a larger study focusing upon malaria transmission, therefore the *An. coustani* complex and *Culex spp* were not identified to species level. The behavioural differences in species scored generally as *Culex spp* might explain the distinctive patterns of relative sensitivity for EGs indoors versus outdoors. The advantage of such an efficacious sampling tool with consistent relative sensitivity indoors and outdoors is even more pronounced in areas where malaria vectors tend to feed outdoors in the early evenings. The apparent ability of EGs to accurately measure P_i_ is the first of two essential steps towards accurate quantification of what proportion of human exposure occurs indoors (π_i_) but the second step is accurate measurement of the proportion of mosquitoes which are caught while most humans are indoors (P_fl_).

Unfortunately, a general observation from this study is that when compared to the HLC gold standard, the EGs tend to decline in relative sensitivity as the night progresses and therefore underestimate the proportion of mosquitoes caught late in the night after most people have gone indoors (P_fl_). Relative sensitivity for the two most abundant *Anopheles* species surveyed declined over time, particularly after midnight for *An. gambiae s.s.* and gradually for *An. coustani*. Although this was not the case for *Culex spp*. caught outdoors, this decline was observed indoors. The difference between these two catching locations may be due to differential levels of endophagy versus exophagy among the variety of species that comprise this taxon so that indoor catches have different taxonomic composition from outdoor catches. The observed decline in sensitivity could also be due to the EGs losing their ability to catch mosquitoes as the night progresses. This phenomenon most probably occurred because battery charge, and therefore voltage, current or capacitance of the grid, dissipated over the course of the night. The lack of data monitoring these properties of the equipment represents a significant limitation of this study. In addition to simple dissipation of battery charge, it has also been suggested that sparks from the EGs might affect mosquito behaviour [33] or that the smell of accumulating numbers of burnt mosquitoes stuck to the EGs might increasingly repel some of the mosquito taxa surveyed here over the course of the night.

**Figure 3:**
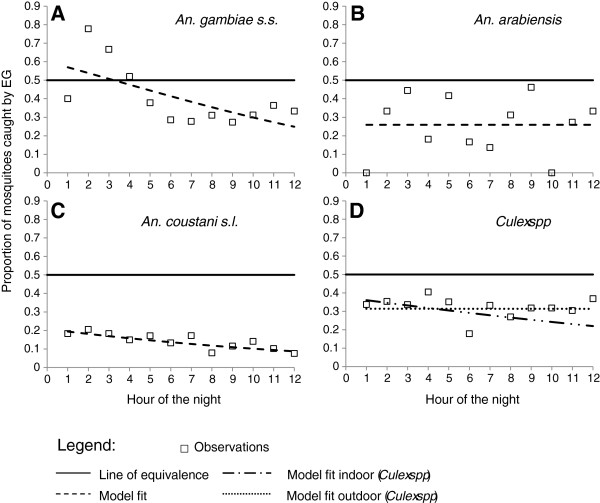
**Sensitivity of EG relative to HLC through the night time.** Relative sensitivity of electrocuting grids to catch *An. gambiae s.s.* (**A**), *An. arabiensis (****B****), An. coustani s.l.* (**C**) and *Culex spp* (**D**) compared to HLC. The plain line assumes equivalence of sensitivity between EG and HLC over night; the squares show the actual observations and the discontinued lines the model fit for the observed values.

The fact that the sensitivity of EGs relative to HLC varied between mosquito taxa, even at the start of the night before any decline occurred (Figure 3), could also be due to a difference in their respective flight behaviour while approaching and departing from the human bait. It would be interesting to test whether mosquitoes in our area have the same flight approach while attacking the host so that they would have the same chances of encountering a physical “barrier” such as the EGs. In previous studies, it has been demonstrated in tunnel experiments that *Cx. quinquefasciatus* and *An. albimanus* behave significantly differently in their approach to their host’s odours*. Cx. quinquefasciatus* fly at a higher speed than *An. albimanus* but conversely turn less degrees in flight. Moreover, *An. albimanus* spends more time in flight than *Cx quinquefasciatus*, therefore increasing the chances to encounter a given barrier for the former than the latter [44].

The EGs used in this study were only 240 mm above the sleeping base, so even slight difference between different species in their flight approach to the human bait might yield significant differences in proportions of mosquitoes caught by the EGs. Because of their low height, they cover only a small proportion of the odour plume emanating from the bait. Therefore, most mosquitoes attempting to reach the person sleeping under the net were flying over rather than through the grid.

Although the commercially manufactured EG traps used in this study clearly underestimated P_fl_ and therefore π_i_, it might be possible to modify them to stabilize trap efficiency and relative sensitivity to address this concern. One way of improving the current design would be to increase the size of the grid frame to maximise the chances of catching mosquitoes as they try to get to the human host.

Another issue for the current grids is the stability of power source and the electrical properties of the grids and supporting circuitry. The EG design used here was not designed by the manufacturer to be used outdoors and was powered by an external car battery connected to an inverter to generate 220V for a set of six connected EGs. It is therefore difficult to be sure that all components of the EG system maintained stable potential, current and capacitance throughout the night as temperatures, humidity, rainfall and battery charge could all vary. Moreover, these devices are cumbersome to carry and move around in the field. A way of improving this would be to design one unit combining the power source and the grid’s frame so that it becomes easy to move them around. The circuits should be designed to run for at least 12 continuous hours with a stable current output to avoid fluctuations through the night.

## Conclusion

The experiments using the EGs described here represent a step closer towards an exposure-free tool that can be readily used for sampling mosquitoes indoors and outdoors and at all times of the night with constant sampling efficacy, relative to HLC. Nevertheless, the current design is not adequate for accurately estimating where and when human exposure to mosquito vectors occurs. This trap design therefore needs to be further improved and evaluated in comparison with HLC so that realistic and practical exposure-free methods can be identified.

## Competing interests

The authors declare that they have no competing interests.

## Author’s contribution

SM participated in the design and supervision of the study, analysed the data and drafted the manuscript, DJM and YM implemented the study and supervised field activities, NJG and SMM contributed to editing the manuscript, DJM, SMM and GFK formulated the hypothesis and experimental objectives, GFK supervised the design of experiments, guided data analysis and contributed to editing the manuscript. All authors read and approved the final version of the manuscript.
